# Types of Intelligence and Academic Performance: A Systematic Review and Meta-Analysis

**DOI:** 10.3390/jintelligence10040123

**Published:** 2022-12-13

**Authors:** Raquel Lozano-Blasco, Alberto Quílez-Robres, Pablo Usán, Carlos Salavera, Raquel Casanovas-López

**Affiliations:** 1 Department of Psychology and Sociology, Faculty of Humanities and Science Education, University of Zaragoza, 50001 Zaragoza, Spain; 2 Department of Educational Sciences, Faculty of Human Sciences and Education, University of Zaragoza, 22003 Huesca, Spain; 3 Department of Psychology and Sociology, Faculty of Education, University of Zaragoza, 50009 Zaragoza, Spain; 4 Department of Educational Sciences, Faculty of Humanities and Science Education, University of Zaragoza, 50001 Zaragoza, Spain; 5 Instituto de Estudios Interdisciplinarios de la Niñez y la Adolescencia (INEINA), Universidad Nacional, Heredia 40101, Costa Rica

**Keywords:** intelligence, academic performance, education, meta-analysis

## Abstract

The concept of intelligence has been extensively studied, undergoing an evolution from a unitary concept to a more elaborate and complex multidimensional one. In addition, several research studies have focused their efforts for decades on the study of intelligence as a predictor of academic performance of students at different educational stages, being a stable and highly relevant predictor along with other variables such as executive functions, social context, culture or parental guardianship. Thus, the present study, based on a systematic review and meta-analysis, includes 27 studies with a total sample of 42,061 individuals. The main objective was to analyse the relationship between intelligence and academic performance using different predictive models that include moderating variables such as country of origin, type of intelligence, gender and age. The findings of this research highlight the significant, positive and moderate relationship between intelligence and academic performance (r = 0.367; *p* < 0.001), highlighting the predictive capacity on school performance when the type of intelligence (general and implicit; 35%) or the country of origin (45%) is taken as a moderating variable, with the explanatory models on age or sex not being significant. Therefore, it can be concluded that intelligence, in addition to being a good predictor of academic performance, is influenced depending on the type of intelligence or theoretical model taken as a reference, and also depending on the country or culture of origin.

## 1. Introduction

The educational community has traditionally and extensively studied academic performance. This concept is closely related to the teaching–learning process focused on a specific goal: achievement in school (Von Stumm and Ackerman 2013). Therefore, issues such as school success or failure, discouragement and dropout have produced a great deal of research (Balkis 2018). Proof of this would be the study by Nieto Martín (2008), who reviewed 654 studies conducted between 1970 and 1990. The author stresses that the variables under study and related to academic success have changed over time; for example, intelligence was traditionally studied from a single-factor point of view, but later this approach was expanded and, at present, other variables such as executive functions, motivation or self-esteem and self-efficacy are at the forefront of the study. In addition, the new century has seen the emergence of new methodological variables such as group collaboration, collaborative work, project-based learning and the length of the school day. The literature has traditionally categorised these variables as contextual or personal: socio-environmental variables (family, friends, colleagues), institutional variables (school, school organisation, teachers) and instructional variables (content, methods, tasks). In addition, another group included are cognitive (intelligence, learning styles) and motivational (self-image, goals, values) variables (Quílez-Robres et al. 2021b). Therefore, academic performance can be understood as a construct that includes quantitative and qualitative values (quantitative if we talk about numerical measurement, test results and qualitative if we talk about the development of skills, values and competences), related to knowledge, attitudes and values developed by the student in the teaching–learning process” (Navarro 2003, pp. 15–16).

However, this study focuses on the relationship and explanation of academic performance through intelligence, understood as different types of intelligence. There is extensive literature on the relationship, prediction and explanation of intelligence with academic performance. However, studies concerning intelligence have expanded conventional psychometric notions by introducing modalities such as crystallised intelligence, fluid intelligence, emotional intelligence, multiple intelligences, etc., in an attempt to provide greater predictive validity in reference to academic achievement (Sternberg 2019).

The conceptual definitions of intelligence are diverse. For (Quílez-Robres et al. 2021a) it consists of the ability to understand and adapt to solve everyday problems. On the other hand, for Plomin and Deary (2015), intelligence has a biological substrate that varies according to individuals and cultures, being the potential that facilitates learning, planning, reasoning, adaptation and decision-making.

Catell (1963) differentiated between two kinds of intelligence: fluid intelligence and crystallised intelligence and suggested that intelligence is composed of different capacities that form general intelligence and that are complementary. Crystallised intelligence is the result of education and culture and therefore depends directly on the individual’s prior knowledge and ability to learn (Nisbett et al. 2012) and fluid intelligence with a genetic component is the ability to solve problems through non-verbal abstract reasoning and adaptation to different contexts (Nisbett et al. 2012). In addition, it is linked to individual learning and memory (Amin et al. 2015). The latter is considered as one of the main predictors of individual academic achievement according to several studies in different settings (Deary et al. 2007; Geary 2011; Laidra et al. 2007; Monir et al. 2016; Verbitskaya et al. 2020). Rabbitt and Lowe (2000) suggest that fluid intelligence is altered in the ageing process, while crystallised intelligence remains stable.

The study of intelligence expanded, and Gardner (1985) proposed an alternative and a critique of the general intelligence approach by elaborating the theory of multiple intelligences. He proposed the existence of several independent intelligences that interact and mutually enhance each other, such as linguistic, logical–mathematical, spatial, kinaesthetic bodily and others. Thus, most students possess more than one. However, Singh et al. (2017) report that only logical–mathematical, spatial and musical intelligence are related to IQ. These results are consistent with the research of Castejon et al. (2010) and Visser et al. (2006), who reported a strong relationship between cognitive component intelligences and general intelligence.

Sternberg (1985) elaborated the Triarchic Theory of intelligence, establishing three categories within it: competency, experiential and contextual. Thus, the acquisition and storage of information, the ability to encode, combine and compare that information and finally the adaptation of information to context were involved. He expanded on this theory and called it successful intelligence, which combined ability, exploitation, adaptation, creativity, etc. It is about being able to solve problems, and depending on the way it is done, analytical intelligence (both familiar and abstract problems), creative intelligence (formulating ideas, problems of a novel nature) and practical intelligence (applying ideas and analysis effectively) will appear (Sternberg et al. 2010).

The theories of Gardner (1985) and Sternberg (1985) were fundamental for the emergence of the theory of emotional intelligence since these ideas underlay the new concept that would germinate in the theories of Salovey and Mayer (1990), but it would be Goleman (1996) who popularised it by stating that emotional intelligence consists of a series of skills such as discovering, recognising and managing emotions and feelings (Goleman 1999). The relevant role of emotional aspects in academic results is evident in previous studies such as the meta-analysis conducted by Molero-Puertas et al. (2020), which concludes with a significant effect size between emotional intelligence and academic performance. Other research suggests that this variable is a good predictor of academic achievement at different educational stages and even indicates that it is second only to general intelligence (MacCann et al. 2020; Perera and DiGiacomo 2013; Sanchez-Ruiz et al. 2013).

Finally, implicit intelligence, regarded as the self-perception of intelligence grounded in everyday experience, is a key variable for understanding academic performance (Enea-Drapeau et al. 2017). Since this includes a component of expectation as cognitive self-representation, some authors point out that the relationship is especially direct in the early years and concerning specific performance areas rather than global performance (Dinger et al. 2013; Geary 2011; Priess-Groben and Hyde 2017; Wigfield et al. 2016); other authors argue that implicit intelligence is a good predictor for academic performance in maths (Kriegbaum et al. 2015; Steinmayr and Spinath 2009); for Steinmayr et al. (2019a) and Lotz et al. (2018), this predictive value extends over all areas, as confidence in one’s own abilities can be a more important variable than cognitive abilities in the analysis of academic performance. In this sense, implicit theories are presented as definitions, or theories that scientists have about some phenomena (Sternberg 1985). Precisely in these beliefs lies the importance of understanding people’s implicit theories. This is important because these beliefs guide people’s attitudes and behaviours and, as discussed in various theories of the development of talent and intelligence, intelligence is not composed of a single factor but is multidimensional, with contextual, creative and motivational aspects related to people’s behaviours intervening in its conception. The theory of social cognition indicates that beliefs determine attitudes and willingness to engage in certain behaviours (Pintrich 2002). Undoubtedly, these aspects mean that implicit intelligence must be taken into account in relation to academic performance and learning.

With regard to academic performance, its prediction has been a relevant topic for a long time and different variables have been analysed to help explain the academic results of schoolchildren. Different research has related it to individual characteristics of basic cognitive processes such as processing speed, working memory, fluid intelligence, etc. (El Jaziz et al. 2020; Kiuru et al. 2012; Kuncel et al. 2004; Richardson et al. 2012; Sternberg et al. 2001). However, academic performance as a product of learning serves as an indicator of the level of learning (Alquichire R and Arrieta R 2018). For Ariza et al. (2018), it is nothing more than a measure of what students have learned as a result of an educational process. He defines it as the ability to respond to a series of educational stimuli, which in turn is interpreted on the basis of the established objectives. 

In view of previous research, it is not new that measures of general intelligence predict academic performance (Deary et al. 2007; Quílez-Robres et al. 2021b; Sternberg 2019). Systematic study has resulted in the predictive value of intelligence in the educational world and has pointed to significant correlations with different variables, but there is also some variation depending on the educational stage analysed (Sternberg et al. 2001). While agreeing that intelligence is one of the most important variables in academic performance as it has a direct impact on learning (González et al. 2008), it should be noted that it does not behave uniformly, as the correlation between intelligence and academic performance decreases when the student reaches the university stage (Ren et al. 2015).

If the aim is to increase the predictive value of the different measures of intelligence, one possibility is to broaden the concept of intelligence itself. A review of the scientific literature shows that there are no studies that integrate the different types of intelligence theorised in reference to academic achievement. This meta-analysis aims to analyse the relationship between different types of intelligence and academic performance from a meta-analytical perspective by reviewing the scientific literature with a broad conception of the concept of intelligence, taking into account the studies that indicate that there is no single way of understanding and defining this construct.

## 2. Method

**Figure 1:**
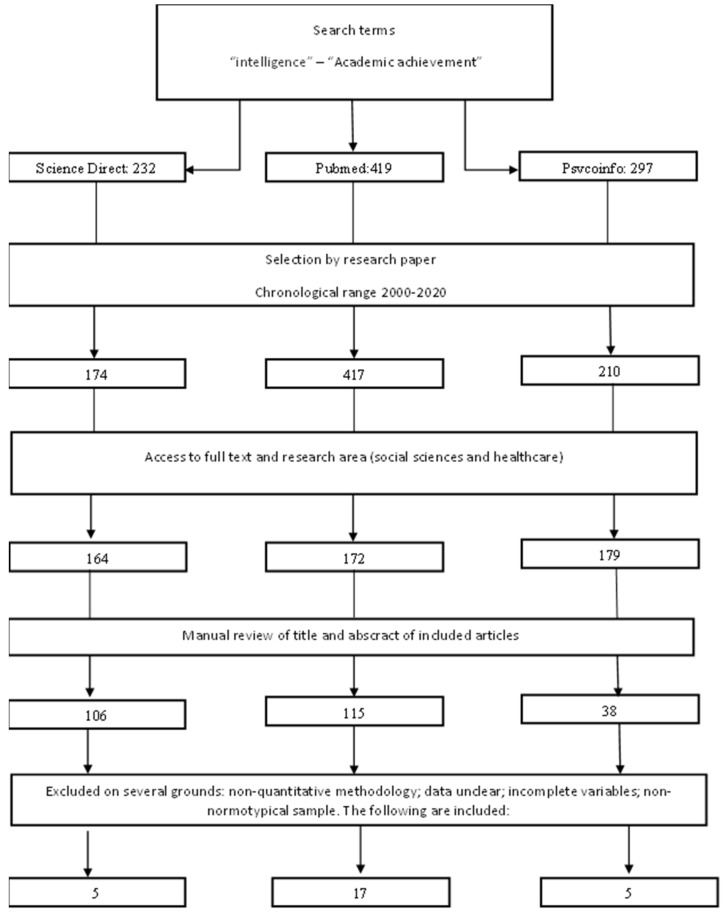
Flowchart of search methodology.

A research registry protocol (Figure 1) was established following the Cochrane systematic review manual in Higgins and Green (2011) and PRISMA (2015). Inclusion criteria were determined using the specifications set out by Ausina and Sánchez-Meca (2015) and Moreau and Gamble (2020): (a) Research methodology: quantitative, correlational, longitudinal, cross-sectional and clinical. (b) Time frame: 2000–2020. (c) Methodological rigour: studies indexed in prestigious rankings (Scimago Journal and Country Rank). (d) Measuring instruments: psychometric tests rated in academic publications and in accordance with the culture of the sample. (e) Language: English.

The exclusion criteria were established according to the manuals of Ausina and Sánchez-Meca (2015) and Moreau and Gamble (2020): (a) Adult population with previous disorders or pathologies, including, however, research in which there were control groups without pathologies. (b) The appearance of imprecise, poorly defined data, unclear methodology or with indications of non-compliance with ethical principles, as well as statistical or psychometric errors in the measurement of the tests, following the indications of Hunter and Schmidt (2004) and Friese and Frankenbach (2020).

The search strategy was carried out using the criteria of Botella and Gambara (2002), Ausina and Sánchez-Meca (2015) and PRISMA (2015). Three databases were used: Psycoinfo, Pubmed and Science Direct, and research was performed in February 2021. The Boleean action was “academic achievement” and “intelligence” in the range 2000–2020.

Eligibility criteria for sample selection were defined according to the Cochrane systematic review manual in Higgins and Green (2011) and PRISMA (2015). It should be noted that manual coding was carried out by reviewing each article returned by Boolean actions according to the inclusion and exclusion criteria. Firstly, the abstract was screened so that only those that dealt with the subject of the study were selected. On the other hand, the criteria of methodological rigour and measurement instruments led to the exclusion of a significant percentage of the research. This was due to the absence of standardised instruments or the incorrect measurement of the study parameters according to the pre-established psychometric test. 

The transformation of all means to Fisher Z (Martín-Andrés and Luna del Castillo 2004), the execution of the relevant analyses (model comparison and meta-regression), the study of heterogeneity, the performance of the Eggers test for publication bias and the obtaining of figures were carried out using the CMA statistical software. 

## 3. Results

### 3.1. Demographic Description

In recent years (2000–2020), the relationship between types of intelligence and academic performance at different educational stages has been studied in depth. In total, the meta-analysis (Table 1) consists of 27 studies with k = 47 samples from Europe, Asia, Africa, America and Oceania. According to Bonett’s (2006) criteria, the sample k = 47 exceeds the minimum required to avoid distortion of the upper confidence limit. On the other hand, heterogeneity is evident in the sample sizes, with the smallest sample size being 81 participants and the largest 4036 participants. 

The total sample is made up of 42,061 participants, 47.16% of whom are male and 48.99% female. In this sense, it is necessary to clarify that the two studies do not provide data on the sex of their participants. The average age of the participants is 16.45 years, although some studies did not report a specific average age, but rather a range of years or school years, making it necessary to take the arithmetic mean to be able to manage the data quantitatively.

In terms of culture, social anthropology points to the need to attend to cultural diversity (Molano 2007). In this study, 30.13% are Asian (China, Indonesia and Malaysia), 4.73% are Central European (Germany, UK), 37.37% are Eastern European (Russia), 6.01% are Northern European (Norway and Finland), 2.32% are North African (Morocco and Egypt), 18.83% are American (USA and Barbados) and 0.57% are from Oceania (Australia). 

### 3.2. Statistical Analysis

The aim of this meta-analysis is to study the relationship between type of intelligence and student achievement, but encompassing different educational stages and different contexts. To this end, 108 effect sizes were coded, taking as a reference the data based on Pearson’s r and their subsequent treatment using the CMA statistical programme.

**Figure 2:**
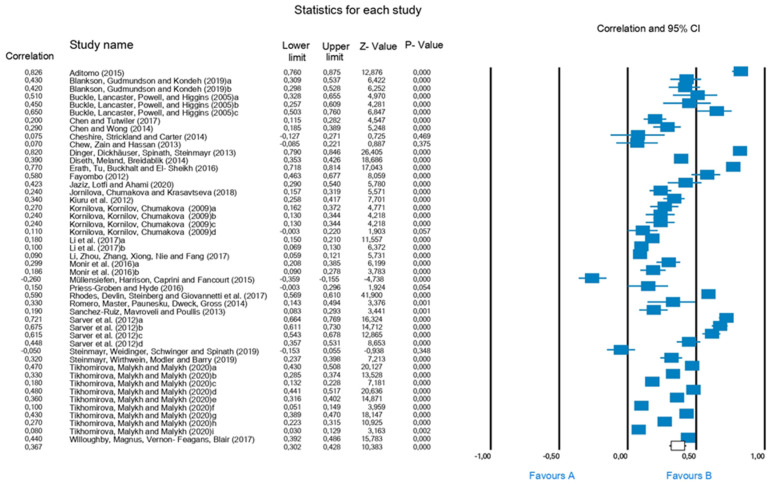
Forest Plot.

Figure 2 (forest plot) shows the effect size with a 99% confidence interval (0.302–0.428, *p* = 0.001) for the different studies, the effect size being r = 0.367, *p* = 0.001. In other words, a moderate level of correlation is obtained according to Cohen between the intelligence presented by the students and academic performance. The ethical criteria set out by Moreau and Gamble (2020) are followed when exposing all the conversions, opting for a policy of “open materials”. 

On the other hand, it is crucial to study the heterogeneity of the sample according to Cochrane in Higgins and Green (2011). The Q statistic of Dersimonian and Laird (1986) (Q = 2478.71, df = 46, *p* < 0.0001) describes a high variability, i.e., the homogeneity hypothesis is rejected. The statistic I2 = 98.144% explains the percentage of variability resulting from heterogeneity and not from chance. In other words, the sample is highly heterogeneous in its statistical nature (Higgins et al. 2013). Consistently, the Random model or random effects model is applied (Bonett 2006; Martín-Andrés and Luna del Castillo 2004). Although the inclusion and exclusion criteria contemplate the reliability and methodological and psychometric quality of the research, the Egg’s test with 99% reliability was carried out to study the effect of bias (Botella and Gambara 2002; Ausina and Sánchez-Meca 2015). The results of the test show the inexistence of publication bias with a 99% confidence interval (*p*-value 1 tailed = 0.07; *p*-value 2 tailed = 0.15) (Egger et al. 1997). The standard error value (SE = 2.04) reaffirms the absence of bias, as it is very close to the regression line (Martín-Andrés and Luna del Castillo 2004). 

**Figure 3:**
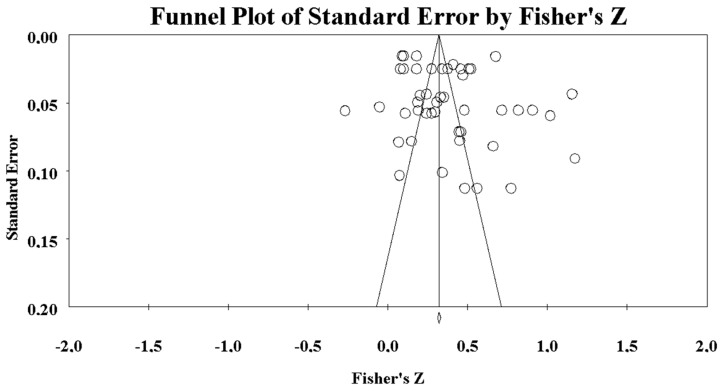
Funnel plot and standard error.

The diversity shown in the Q and I2 statistics could be a sign of extreme data; however, the tight confidence interval (0.302–0.428, *p* = 0.001) limits this heterogeneity. These results are consistent with the Funnel Plot graph (Figure 3) where the variability and heterogeneity of the sample are reaffirmed. This situation reiterates the diversity of studies, as concluded by the Egger test, without any bias effect. However, it should be noted that the apparent variability could be affected by the transformation to Fisher *Z*-values since x-values >0.5 tend to be more distorted on the T-Student curve than, in comparison, on the normal curve, although this transformation is accepted by the scientific community for meta-analysis methodology (Martín-Andrés and Luna del Castillo 2004).

### 3.3. Moderating Variables and Meta-Regression Analysis

The state-of-the-art research shows the existence of moderating factors, which is why it is considered necessary to establish the study of seven moderating variables: type of intelligence, type of performance, age, country, male sex, female sex and geographical distribution. The objective pursued through the use of both techniques is to statistically determine the reason for such heterogeneity (Ausina and Sánchez-Meca 2015; Jak and Cheung 2020). In this way, a comparison of models is established (see Table 2) by generating seven models: (1) type of intelligence; (2) type of performance; (3) age; (4) country; (5) male gender; (6) female gender; (7) geographical distribution. 

The first model, which specifies the type of intelligence, explains 35% of academic performance, with an efficiency level of over 99%, although, as model 2 shows, the type of performance has no effect. In other words, it is intelligence that determines student academic performance and success, but doing well in these subjects does not seem to affect intelligence overall. On the other hand, the models of age, gender and geographical distribution do not explain the relationship between the two factors to any extent. However, there are important differences between countries, which may be explained by diversity in the education system. Association with a given nation accounts for 45% of the variability in the sample (Table 2).

It is therefore necessary to study in greater depth the type of intelligence that seems to determine academic performance. For this reason, a meta-regression (Table 3) is carried out in which it is evident that general intelligence (Z = 2.00, *p* = 0.04) and implicit intelligence (Z = 3.69, *p* = 0.00) are the ones that stand out, showing a clear difference. 

As far as the different countries are concerned, significant differences are found in the comparison models. Therefore, it is necessary to perform a meta-regression (see Table 4) that points out the differences between education systems. In this case, the countries that differ from the sample are China, Indonesia and the UK (United Kingdom). 

## 4. Discussion

Given that the review of the scientific literature indicates that there is no single way of understanding, defining and analysing the construct of intelligence, this meta-analysis analyses the relationship between intelligence and academic performance in terms of the different types of intelligence studied in previous research, as well as the existence of models of moderating variables that clarify their predictive nature. Therefore, effect size, type of intelligence (general, crystallised, fluid, implicit, emotional, etc.), age, gender, country of residence or geographical area are of interest for this study. Of all these variables, effect size, general intelligence, implicit intelligence (R^2^ = 0.35; *p* < 0.001) and country of residence (R^2^ = 0.45; *p* < 0.001) are those that appear to be relevant and significant.

From the results obtained, a number of factors stand out, such as the relationship between academic performance and intelligence with a moderate effect size (0.367; significance < 0.001). Previous research addressing the interrelations between intelligence and academic performance indicates that it is the most stable and powerful predictor of school performance (r = 0.5) (Geary 2011; Laidra et al. 2007; Luo et al. 2006; Rodic et al. 2015). These results are corroborated in the meta-analysis of Cortés Pascual et al. (2019) who equate it with that obtained for executive functions. They point out that intelligence is decisive for new learning and, on the contrary, executive functions are primordial for repetitive and competence-focused learning and also show their relationship in different educational disciplines. 

Another noteworthy element of the research is that when analysing moderating variables and comparing models, it is found that intelligence determines that the relationship with academic performance is unidirectional. That is, intelligence is a good predictor of academic achievement, but not the other way around, so the predictive model of intelligence type explains 35% of the variance. Consistent with this result, Buckle et al. (2005) assigned it a predictive power of 26%. This is in line with previous research findings that intelligence is the best predictor of academic success (Blankson et al. 2019; Erath et al. 2015; Li et al. 2017; Quílez-Robres et al. 2021a; Ren et al. 2015; Rhodes et al. 2017; Tikhomirova et al. 2020). However, most studies have related it to the cognitive dimension (Castejon et al. 2010; Visser et al. 2006), marginalising the behavioural and emotional aspects (Gioia et al. 2017). Therefore, it is necessary to consider other facets of intelligence, as they are nothing more than different capacities that complement each other (Catell 1963). 

From the meta-regression of the intelligence model, general and implicit intelligence emerge with significance (*p* < 0.05 and *p* < 0.01). Implicit intelligence is decisive in school outcomes, as the beliefs that are elaborated about one’s own intelligence and the nature of intelligence guide student behaviours towards achieving success or failure at school (Chen and Tutwiler 2017; Lotz et al. 2018; Steinmayr et al. 2019b). Thus, it is considered relevant for its efficacy in considering that cognitive ability is not a fixed trait but has an adaptive quality that gives it incremental strength. This malleability performs a protective function against school failure, as there is confidence in one’s own abilities. However, it seems that this incremental capacity decreases with age (Chen and Tutwiler 2017). In the same line of research, Kornilova et al. (2009) found that implicit intelligence predicts general intelligence, as by adopting learning goals and increasing their competence, students overcome setbacks and seek new challenges. It should also be noted that both general and implicit intelligence show an indirect effect with academic success through other variables such as motivation or executive functions (Aditomo 2015). Furthermore, it should be noted that general intelligence has traditionally been broken down into fluid and crystallised intelligence. “Crystallised” intelligence has been considered one of the most significant predictors of individual achievement in different contexts, age ranges and educational conditions (Deary et al. 2007; Nisbett et al. 2012; Verbitskaya et al. 2020), and “fluid” intelligence has been found to be a better predictor of processing speed tests (Luo et al. 2006) and mathematics performance (Blankson et al. 2019; Sarver et al. 2012).

Neither emotional intelligence nor the different types of multiple intelligences show remarkable values in this research. In this regard, the scientific literature is not conclusive. Some studies find that emotional intelligence occupies a pre-eminent position behind general or global intelligence (MacCann et al. 2020; Perera and DiGiacomo 2013) and explain that this type of intelligence is related to academic performance due to its importance in promoting adaptive behaviours (Chew et al. 2013; Fayombo 2012; Usán Supervía and Quílez Robres 2021). The perception of positive interpersonal and intrapersonal emotional intelligence substantially explains academic success, as it comprises learner ability to control, regulate and manage the demands of the academic context (Cheshire et al. 2015; Chew et al. 2013; Kornilova et al. 2018; Okwuduba et al. 2021; Romero et al. 2014). However, the research that studies this claim presents mixed results since some authors such as Engin (2017) or Zhoc et al. (2018) did not observe associations between academic performance and emotional intelligence. On the other hand, and within the multiple intelligences, musical intelligence has been related to academic performance, especially through cross-sectional data, from which it is difficult to infer a generalisation of cause and effect with respect to school achievement (Müllensiefen et al. 2015; Schellenberg 2011). This is despite the fact that Castejon et al. (2010) and Visser et al. (2006) point to the existence of a relationship between some of the multiple intelligences and general intelligence due to their cognitive component.

Concerning the type of “perfomance”, comparison analysis model showed its non-significance. However, it is necessary to point out that the performance types are not homogeneous in the meta-sample. There is a large amount of general performance, but there are hardly any cases of music or mathematics. Despite its non-significance, investigation of this aspect in further research is considered necessary, and nevertheless, there are studies that advocate the importance of this variable.

As for gender and age, they are not moderating variables, perhaps influenced by the type of assessment of these variables and the different theoretical concepts of greater or lesser importance assigned to the relationship between them. On the other hand, there are difficulties in predicting the role of gender and age in implicit intelligence (Diseth et al. 2014), but Robins and Trzesniewski (2005) point out that there is a strong relationship in favour of girls and at an older age. These results can be related to emotional intelligence and higher perceived self-efficacy (King et al. 2012).

As noted above, the country of residence model is the moderating variable that explains 45% of the variance, increasing the predictive power of the intelligence type model. These results are consistent with previous research pointing to the importance of adaptation to different contexts (Deary et al. 2007; Verbitskaya et al. 2020) or those indicating that the relationship between intelligence and academic performance was the result of education and the culture in which one was immersed (Nisbett et al. 2012; Plomin and Deary 2015; Rodic et al. 2015). In his theories, Sternberg, for example, pointed out the importance of adaptation to the context of different skills and abilities, as well as of the differences originating in the beliefs of one’s own abilities in their contribution to academic achievement as a function of the cultural environment that generate individual profiles with different strengths and weaknesses (Sternberg 2019; Sternberg et al. 2001). Ultimately, intelligence is related to social competence (Sternberg 1985).

When analysing the meta-regression across countries, three countries are significant: Indonesia, the United Kingdom (UK) and China. Indonesia is considered a very deterministic culture (if you are not very smart, you do not pass) (Aditomo 2015). On the other hand, the United Kingdom (UK) as a model of the Anglo-Saxon education system associates intelligence with linguistic ability and problem-solving skills (Sternberg 1985). Finally, in China, authoritarian filial piety beliefs are associated with an entity view of intelligence, which impairs the students’ academic performance (Chen and Wong 2014). Cultural views of motivational processes can shed light on the ways in which motivational beliefs develop as a product of cultural or socialisation processes, which, in turn, contribute to or determine the students’ academic success (Chen and Wong 2014; Li et al. 2017). These differences by country of origin are likely to point to the meanings attributed to intelligence by different cultural groups. There are indications that individuals from Western countries attach a much broader meaning to the concept of intelligence (skills, context, etc.) and, therefore, when studying subjects from non-Western countries, consideration should be given to using specific domains that provide greater certainty to the results, always bearing in mind that the mindset about intelligence and academic ability is very different (Aditomo 2015). As Carroll (1992) points out, intelligence is a concept within the mind of a society and personal references are those of each culture where individuals are immersed. Some cultures such as the Asian ones continue to use teaching–learning methods based on cognitive aspects such as memory and one’s own intelligence, while the European and Anglo-Saxon models are based on the development of competence through social interaction (Quílez-Robres et al. 2021a).

Other reason may be due to different factors such as, for example, the statistical weight of the samples, or others related to cultural elements such as different understandings of academic performance and different assessments of different types of intelligence.

Furthermore, following Serpell (2000), culture can be approached from three perspectives: culture as a language, culture as a womb, and culture as a forum. According to the language perspective, culture would constitute a distinct system of meanings in the mind within which the concept of intelligence would be embedded. According to the womb perspective, human cultures create environments that nurture personal growth and stimulate the development of human intelligence. Finally, the forum view, which is based on the interaction of members of a community organising aspects of education and constructing new meanings about intelligence, proposes research on cognitive development as a function of culture.

On the other hand, Sternberg and Grigorenko (2004) indicate that intelligence cannot be understood completely outside of cultural control or influence. There are behaviours that are considered intelligent in some cultures, and those same behaviours are considered unintelligent in other cultures. Furthermore, each culture has implicit (folk) theories of intelligence, and therefore the aspects that fall under this concept vary from culture to culture. In this sense, the three influential cultures in this study belong to two different cultural approaches: individualistic (UK) versus collectivistic (China and Indonesia). Moreover, these countries have different ways of understanding academic performance and attach different degrees of importance to intelligence in academic, social and occupational performance (Quílez-Robres et al. 2021b).

## 5. Conclusions

This research was conducted to identify the ways in which different aspects of student intelligence contribute to differences in academic performance. Of the seven models studied, the country of residence model was found to be the most important predictor of academic performance, explaining 45% of the variance, followed by the type of intelligence model, which explains 35% of the variance. The latter model highlights the importance of general intelligence and implicit intelligence for student grades in academic subjects. The results therefore extend knowledge about the role of intelligence for academic achievement. Implicit intelligence scores better in relation to academic achievement than global intelligence, highlighting the importance of one’s beliefs in one’s own abilities. Students with similar intelligence scores, with identical values and the same prior attainment will see improved academic outcomes by believing in their own competencies and abilities (Steinmayr et al. 2019a). If one concludes that academic performance is determined by a multitude of variables including psychological factors that influence student response to overcome setbacks, the evidence points to intelligence as a predictor of success, but also, as this research shows, to a positive mindset in relation to one’s own intelligence and academic abilities. This positive mindset will also be established by the context in which their academic life takes place, i.e., society, beliefs, values, education system, etc. (Aditomo 2015; Hong et al. 1999). Therefore, the results of this study point the way to implement interventions aimed at improving the students’ own beliefs about their subject-specific mastery skills.

Finally, we conclude with the need to expand the study in order to limit the term intelligence. What would its general structure be, and how do the different types of intelligence add significance to the general and traditional concept? What conceptual divergences exist between the different theories? Do all these concepts have the same impact on new or repeated learning, on general and specific?

## Figures and Tables

**Table 1:** Sociodemographic data.

Authors	Number of Samples	Size of Samples	Age	Female	Male	Type of Intelligence	Type of Achievement	Country	Geographical Region
Aditomo (2015)	1	123	18.67	99	24	general	general	Indonesia	Asia
Blankson et al. (2019)	2	198	4.84	106	92	fluid	mathematics	USA	North America
Buckle et al. (2005)	3	81	16.02	41	40	general	general	Australia	Oceania
Chen and Tutwiler (2017)	1	506	11	247	259	emotional	general	USA	North America
Chen and Wong (2014)	1	312	19.88	187	125	general	general	China	Asia
Cheshire et al. (2015)	1	96	21.46	71	11	emotional	general	USA	North America
Chew et al. (2013)	1	163	21.8	112	51	emotional	general	Malaysia	Asia
Dinger et al. (2013)	1	524	17.43	278	246	implicit	general	Germany	Central Europe
Diseth et al. (2014)	1	2062	12	1031	1031	emotional	general	Norway	Northern Europe
Erath et al. (2015)	1	282	10.4	154	126	general	general	USA	North America
Fayombo (2012)	1	151	22.8	88	63	emotional	general	Barbados	America
El Jaziz et al. (2020)	1	167	16.34	95	72	kinesthetic	general	Morocco	North Africa
Kornilova et al. (2018)	1	521	20.56	374	147	emotional	general	Russia	Eastern Europe
Kiuru et al. (2012)	1	476	13	290	186	general	general	Finlandia	Northern Europe
Kornilova et al. (2009)	4	300	19.48	221	79	general	general	Russia	Eastern Europe
Li et al. (2017)	3	4036	15.41	2066	1970	emotional	general	China	Asia
Monir et al. (2016)	2	407	9.5	203	204	general	lenguage	Egypt	North Africa
Müllensiefen et al. (2015)	1	320	14.14	No data	No data	general	general	UK	Central Europe
Priess-Groben and Hyde (2017)	1	165	17.35	77	88	implicit	general	USA	North America
Rhodes et al. (2017)	1	3826	71.19	2066	1760	general	general	USA	North America
Romero et al. (2014)	1	115	12.70	67	48	emotional	general	USA	North America
Sanchez-Ruiz et al. (2013)	1	323	23	113	210	emotional	general	UK	Central Europe
Sarver et al. (2012)	4	325	10.67	146	179	general	musical	USA	North America
Steinmayr et al. (2019a)	1	354	17.48	200	145	verbal	general	Germany	Central Europe
Steinmayr et al. (2019b)	1	476	16.43	244	232	general	general	Germany	Central Europe
Tikhomirova et al. (2020)	9	1560	6.8	718	842	fluid	language	Russia	Eastern Europe
Willoughby et al. (2017)	1	1120	4	No data	No data	general	general	USA	North America

**Table 2:** Comparison of models: Random effects (MM), Z distribution, Fisher’s Z.

Models	TauSq	R²	Q	df	*p*-Value
Model 1 Intelligence	0.03	0.35	30.49	9	0.0004
Model 2 Performance	0.06	0.00	6.33	5	0.2758
Model 3 Age	0.05	0.05	0,00	1	0.9754
Model 4 Country	0.03	0.45	54.65	12	0.0000
Model 5 Female	0.06	0.00	2.81	1	0.09
Model 6 Male	0.05	0.03	2.92	1	0.08
Model 7 Geography	0.03	0.37	15.73	1	0.15

**Table 3:** Meta-regression of model 1: Intelligence.

Meta-Regression M.1									
Covariate	Coefficient	Standard Error	95% Lower	95% Upper	*Z*	2-Sided *p*-Value	Q	df	*p*
Intercept	0.10	0.20	−0.29	0.49	0.50	0.61	30.49	9	0.0004
Crystallised	0.34	0.29	−0.22	0.91	1.19	0.23			
Emotional	0.13	0.21	−0.27	0.55	0.64	0.52			
Spatial	0.01	0.28	−0.55	0.57	0.04	0.97			
Fluid	0.24	0.20	−0.17	0.65	1.15	0.25			
General	0.41	0.20	0.08	0.82	2.00	0.04			
Implicit	1.05	0.28	0.49	1.61	3.69	0.00			
Mathematical	0.14	0.28	−0.42	0.71	0.50	0.61			
Synaesthetic	0.35	0.29	−0.22	0.92	1.20	0.23			
Verbal	0.19	0.23	−0.26	0.65	0.82	0.41			

**Table 4:** Meta-regression of model 2: countries.

Meta-Regression M.2									
Covariate	Coefficient	Standard Error	95% Lower	95% Upper	*Z*-Value	2-Sided *p*-Value	Q	df	*p*
Intercept	0.48	0.10	0.26	0.69	4.42	0.00	54.65	12	0.0000
Australia	0.12	0.16	−0.20	0.44	0.74	0.45			
Barbados	0.17	0.22	−0.27	0.62	0.78	0.43			
China	−0.31	0.14	−0.60	−0.03	−2.22	0.02			
Egypt	−0.23	0.17	−0.57	0.10	−1.36	0.17			
Finland	−0.13	0.21	−0.55	0.29	−0.59	0.55			
Indonesia	0.69	0.23	0.23	1.14	2.97	0.00			
Malaysia	−0.41	0.22	−0.86	0.03	−1.82	0.06			
Morocco	−0.32	0.22	−0.47	0.41	−0.14	0.88			
Norway	−0.07	0.21	−0.49	0.34	−0.34	0.73			
Russia	−0.19	0.12	−0.43	0.03	−1.65	0.09			
UK	−0.52	0.17	−0.86	−0.17	−2.98	0.00			
USA	0.09	0.12	−0.14	0.33	0.76	0.44			
